# Oscillating Magnetic Field Induced Bone Injury Repair by using Drug‐Free Micromotors

**DOI:** 10.1002/advs.202503254

**Published:** 2025-06-29

**Authors:** Jie Shen, Rui He, Jiajun He, Lipeng Liao, Yongcan Huang, Shaoxiong Min, Xiaoreng Feng, Bin Chen, Ben Wang

**Affiliations:** ^1^ Shenzhen Key Laboratory of Spine Surgery Department of Spine Surgery Peking University Shenzhen Hospital Shenzhen 518036 P. R. China; ^2^ Department of Spine Surgery Peking University Shenzhen Hospital PKU‐Shenzhen Clinical Institute of Shantou University Medical College Shenzhen 518036 P. R. China; ^3^ College of Chemistry and Environmental Engineering Shenzhen University Shenzhen 518055 P. R. China; ^4^ Division of Orthopaedics and Traumatology Department of Orthopaedics Nanfang Hospital Southern Medical University Guangzhou 510515 P. R. China

**Keywords:** bone injury repair, hydrogel, magnetic actuation, micromotors, miniature robots

## Abstract

Bone injury repair remains a significant clinical challenge due to the tissue's limited self‐healing capacity and the complex physiological environment at the defect site. Factors such as insufficient vascularization, poor retention of therapeutic agents, and the lack of effective mechanical stimulation further hinder the success of current minimally invasive treatments, which often rely on the delivery of drugs or stem cells. Here, magnetic gelatin/hyaluronic acid composite hydrogels micromotors are developed, capable of promoting bone regeneration through localized micromovement stimulation, eliminating the need for therapeutic payloads. By harnessing the mechanical forces generated by the micromotors under an oscillating magnetic field, the approach directly enhances osteoblast proliferation and differentiation, providing a novel mechanism for bone repair. The efficacy of this strategy is further validated in vivo using animal models of bone defects, where moderate micromovement stimulation is shown to significantly increase the volume fraction of newly formed bone by approximately twofold, accompanied by well‐aligned collagen and organized mineralization, thereby demonstrating substantial regenerative effects. This work presents a paradigm shift in bone repair with a payload‐free, minimally invasive solution that overcomes conventional limitations and offers new insights into microrobotics in regenerative medicine.

## Graphical abstract

This work presents a payload‐free, minimally invasive approach for bone repair using magnetic hydrogel micromotors, which enhance bone regeneration via localized micromovement stimulation, achieving a twofold increase in new bone formation in vivo.

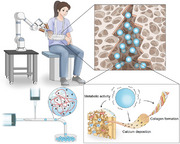

## 1 Introduction

Bone injuries represent a significant clinical challenge due to the tissue's limited self‐healing capacity, poor vascularization, and the complex physiological environment at the defect site.^[^ ^1^ ^]^ Unlike soft tissues, bone defects, particularly critical‐sized ones, often fail to regenerate without external intervention, leading to long‐term functional impairment and increased risk of complications.^[^ ^2^ ^]^ Current therapeutic approaches, including bone grafting, biomaterial scaffolds, and pharmacological treatments, struggle with challenges such as insufficient vascular integration, inadequate mechanical stimulation to promote cellular activity, and the difficulty of achieving localized and sustained delivery of therapeutic agents in the dynamic physiological environment of bone defects.^[^ ^3^, ^4^ ^]^ Furthermore, many existing treatments are invasive, rely heavily on external grafts or bioactive agents, and often fail to provide the precise control needed to stimulate and guide bone regeneration effectively.^[^ ^5^ ^]^ Therefore, there is a critical demand for minimally invasive solutions that not only address these limitations but also directly enhance the biological and mechanical conditions necessary for efficient and reliable bone healing.

Microrobotics has emerged as a transformative field in regenerative medicine, offering precise control, targeted delivery, and minimally invasive therapeutic capabilities.^[^ ^6^, ^7^ ^]^ Among these, magnetic microrobots have attracted significant attention due to their ability to navigate and operate in complex physiological environments under external magnetic fields.^[^ ^8^, ^9^ ^]^ Previous studies have primarily focused on using microrobots to deliver therapeutic payloads, such as stem cells, growth factors, or drugs, to the site of injury.^[^ ^10^, ^11^, ^12^, ^13^, ^14^, ^15^, ^16^, ^17^, ^18^, ^19^, ^20^, ^21^, ^22^, ^23^, ^24^ ^]^ For instance, Choi et al.^[^ ^25^, ^26^, ^27^ ^]^ developed magnetic microrobots loaded with human adipose‐derived mesenchymal stem cells for localized tissue regeneration. This system utilized a fixed magnetic ring to anchor the microrobots at the site of injury, ensuring stable positioning even in challenging physiological environments. The study demonstrated promising results in a rabbit model, highlighting the potential of microrobots to enhance the precision and efficacy of regenerative therapies. However, most existing approaches rely heavily on the retention and stability of therapeutic agents at the defect site, which can be compromised by physiological challenges such as fluid flow or inadequate mechanical stimulation.^[^ ^28^ ^]^ Furthermore, these approaches often overlook the potential of microrobots themselves to actively contribute to tissue regeneration through mechanical interactions, an area that remains underexplored in bone repair.

**Scheme 1:**
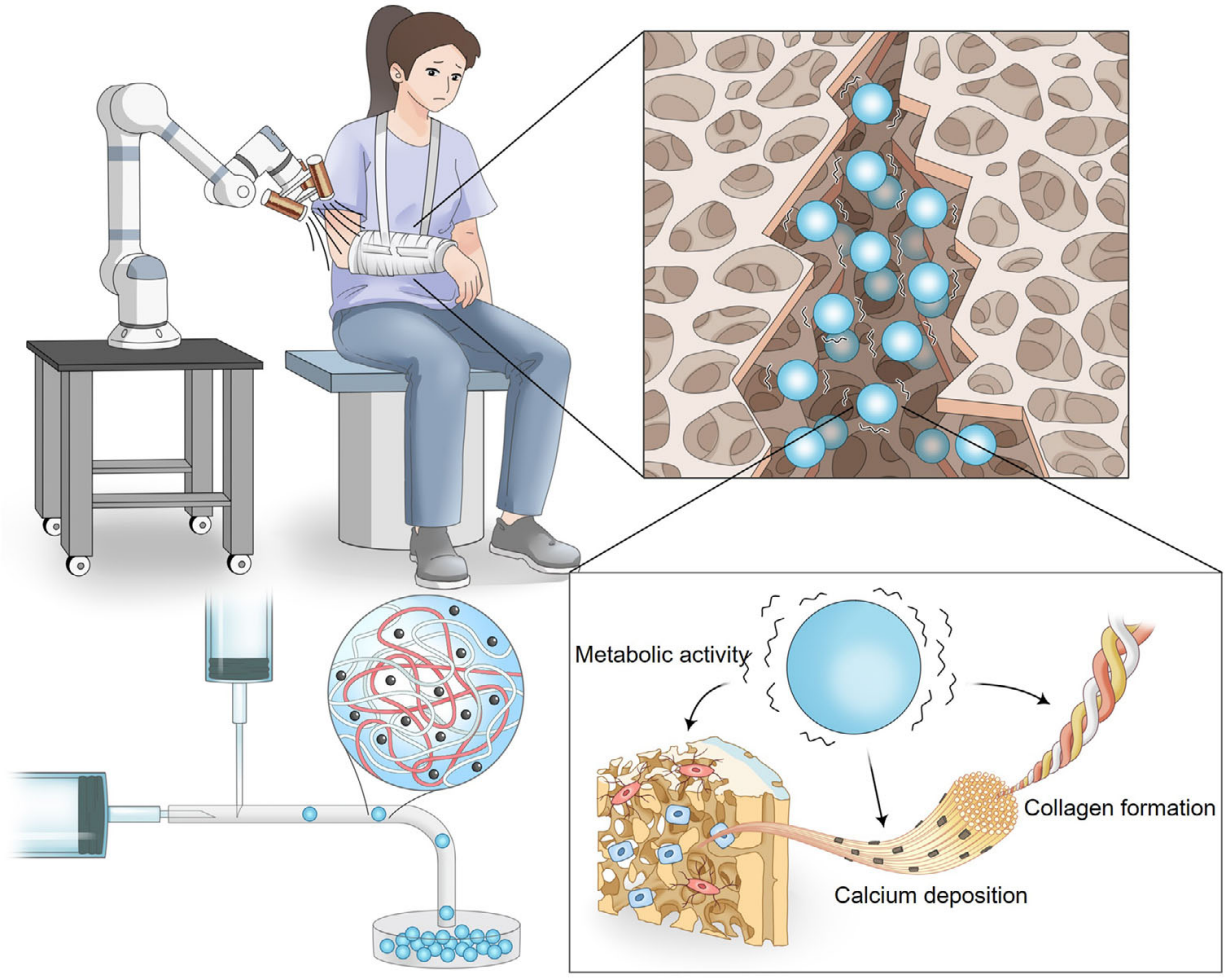
Development of micromotion‐based magnetic hydrogel micromotors (MMHMs) for controlled bone defect repair.

In this study, we present a novel approach to bone injury repair by leveraging the micromovement capabilities of biocompatible magnetic hydrogel micromotors (**Scheme** **1**). Unlike conventional strategies that depend on delivering therapeutic payloads, our work demonstrates that these micromotors can directly stimulate bone regeneration through localized mechanical forces, providing a payload‐free and minimally invasive solution. The micromotors, constructed from biocompatible and biodegradable materials, are driven by an oscillating magnetic field to generate controlled micromovements, which enhance osteoblast proliferation, differentiation, and matrix mineralization. This innovation represents a novel advancement in bone repair by utilizing microrobots as active stimulators of regeneration, rather than passive delivery vehicles. While previous studies have explored mechanical stimulation strategies, such as piezoelectric scaffolds,^[^ ^29^ ^]^ these approaches rely on static or externally powered mechanisms. In contrast, our microrobotic system achieves dynamic, localized micromovements under magnetic actuation, offering precise control over the spatiotemporal application of mechanical forces in a minimally invasive manner. The efficacy of this approach was validated through a series of in vitro and in vivo experiments. In a rat femoral defect model, moderate micromovement stimulation by the micromotors significantly increased the volume fraction of newly formed bone by approximately twofold, with well‐aligned collagen and organized mineralization. Unlike conventional mechanical stimulation therapies such as ultrasound and vibration, which deliver broad, non‐specific stimulation to the treatment area, our approach leverages biocompatible magnetic hydrogel micromotors (MMHMs) to provide localized, programmable micromotion at the defect site. By addressing the challenges of therapeutic retention, stability, and mechanical stimulation in bone repair, this study not only expands the scope of microrobotics in regenerative medicine but also offers a transformative, payload‐free solution for overcoming the limitations of conventional bone repair strategies.

## 2 Results and Discussion

### 2.1 Preparation and Characterization of MMHMs

**Figure 1:**
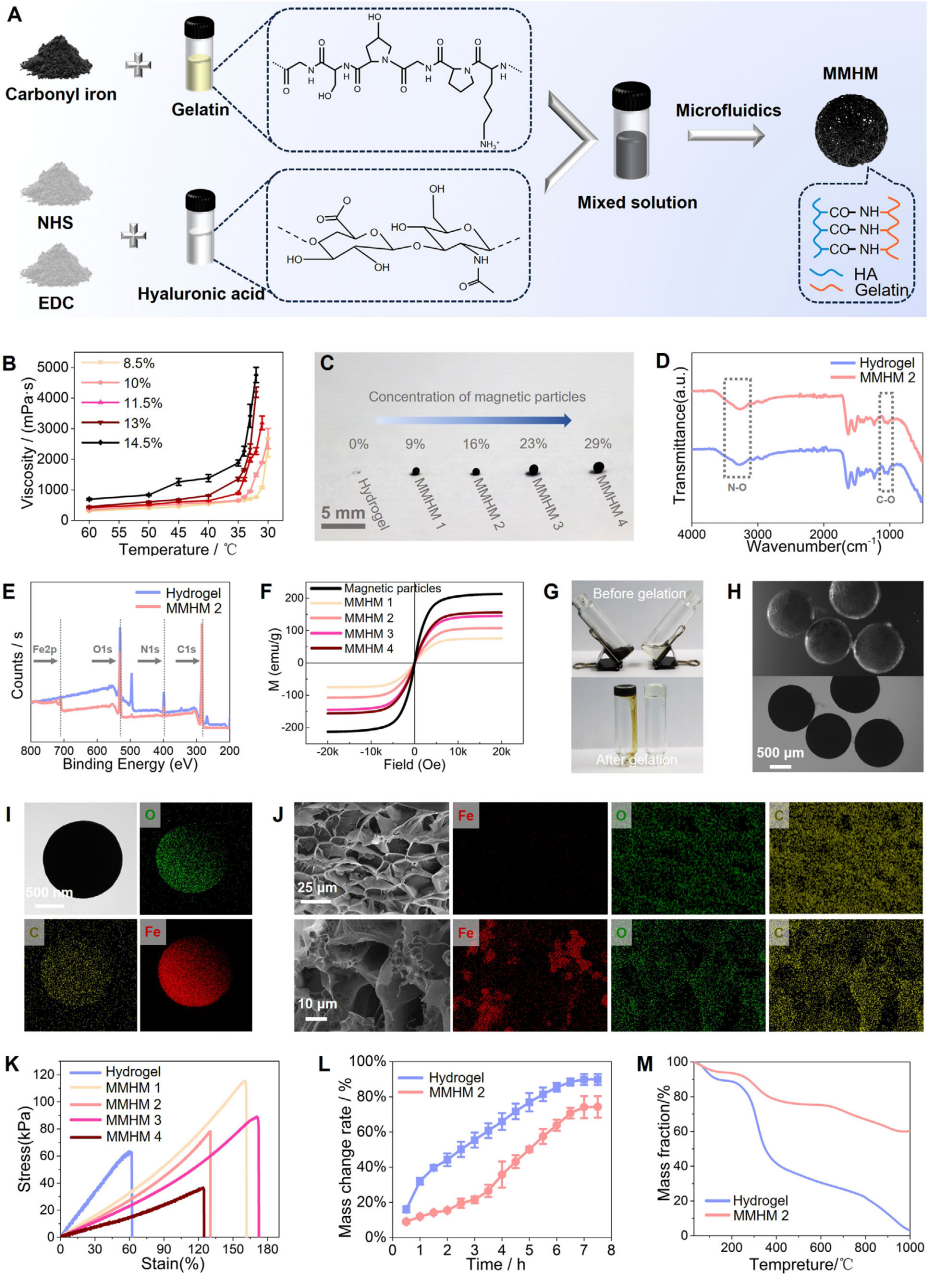
Fabrication and characterization of MMHMs. A) Preparation process of MMHMs. B) Viscosity‐temperature curves of composite hydrogels with different concentrations of gelatin (the mass ratio of hyaluronic acid to gelatin in all samples was 1:15). C) Photograph of pure hydrogel and MMHMs with varying concentrations of magnetic particles. D) Fourier infrared spectra of pure hydrogel and MMHM 2. E) X‐ray electron spectra of pure hydrogel and MMHM 2. F) Hysteresis loops of pure magnetic particles and MMHMs with different concentrations of magnetic particles. G) Photographs of gelation transition of pure hydrogel and magnetic hydrogel. H) Optical microscope images of MMHMs. I) Transmission electron microscopic observation and element maps of carbonyl iron particle. J) Scanning electron microscope images and Energy‐dispersive X‐ray spectroscopy maps of pure hydrogel (upper) and MMHM (lower). K) Mechanical properties of pure hydrogel and MMHMs with different concentrations of magnetic particles. L) Water absorption of pure hydrogel and MMHM 2. M) Thermogravimetric analysis of pure hydrogel and MMHM 2.

Gelatin and hyaluronic acid were used as hydrogel matrices, and cross‐linking was achieved through NHS/EDC‐mediated amidation reaction to form a 3D network structure, ensuring biocompatibility and adjustable mechanical properties. The higher the concentration of the composite hydrogel, the higher the viscosity of its solution (**Figure** **1**B). To take into account both the mechanical properties and processibilities of the material, a composite hydrogel with a mass fraction of 10% gelatin and 1.33% hyaluronic acid was used. Carbonyl iron was introduced as magnetic particles, and microfluidics technology was used to achieve uniform dispersion of particles in the gel to form micromotion‐based magnetic hydrogel micromotors (MMHM 1–4, Figure 1A,C). The particle concentration gradient (0–29%) was accurately controlled to optimize magnetic responsiveness and structural uniformity. Using microfluidic technology, the flow rate ratio of the water phase and the oil phase can be adjusted to prepare hydrogel microspheres of different sizes while maintaining good uniformity. To prepare the MMHMs, the effect of the flow rate ratio of different phases on the size of the microspheres in microfluidic platform was investigated. At 37 °C, when the flow rate ratio of the oil phase to the water phase was 300:10, the micromotor with a carbonyl iron powder content of 16% were prepared with a diameter of 900 µm, while when the flow rate ratio was 300:5, the diameter was 1.5 mm.

The composition of the hydrogel was first characterized by Fourier transform infrared spectroscopy. During the preparation of the hydrogel, the addition of the cross‐linker activated the carboxyl groups in the hyaluronic acid and reacted with the amino groups in the gelatin to undergo an amidation reaction. Macroscopic sol–gel transition process (Figure 1G) and characteristic peaks at 3277.9, 1630.2, and 1000–1500 cm^−1^ confirmed this process (Figure 1D). The X‐ray photoelectron spectrometer (XPS) full spectra of pure hydrogel and MMHM 2 are shown in Figure 1E. The Fe2p peak of the MMHM 2 indicates that carbonyl iron is successfully combined with hydrogel. The high overlap of C1s, O1s, and N1s peaks of the two curves demonstrates that the incorporation of carbonyl iron has no effect on the structure of the hydrogel.

Within the range of magnetic field strength, the magnetization intensity of the MMHM series samples increased significantly with the increase of magnetic particle concentration (Figure 1F). The 29% concentration sample (MMHM 4) showed the highest saturation magnetization intensity (153 emu g^−1^), which is consistent with superparamagnetic behavior. It can be seen that the size and shape of the hydrogel microspheres prepared by the microfluidic platform are uniform (Figure 1H). Scanning electron microscope (SEM) images showed that the hydrogel microspheres have a porous structure, and carbonyl iron particles with a particle size of about 1.3 µm are distributed within these porous structures of the MMHM 2 (Figure 1I,J). The addition of carbonyl iron powder reduced the Young's modulus and tensile limit of the hydrogel (Figure 1K). As the concentration of magnetic particles increases, the Young's modulus of the materials decreased significantly, from 56 kPa for MMHM 1 to 26 kPa for MMHM 4. Although hydrogels containing high concentrations of carbonyl iron may have better responses under magnetic fields, their low mechanical strength may lead to structural instability in usage scenarios. The water absorption of the cross‐linked hydrogels and the MMHM 2 was studied by soaking the different hydrogels in water (Figure 1L). Both hydrogels maintained their water absorption behavior for 7 h and eventually increased their mass by 80% and 65%. For the thermal stability of the materials, both the pure hydrogel and MMHM 2 degraded rapidly in the range of 250–350 °C (Figure 1M). At 1000 °C, the pure hydrogel was almost completely degraded, while MMHM 2 retained 60% of its mass.

### 2.2 Motion Control of MMHMs

**Figure 2:**
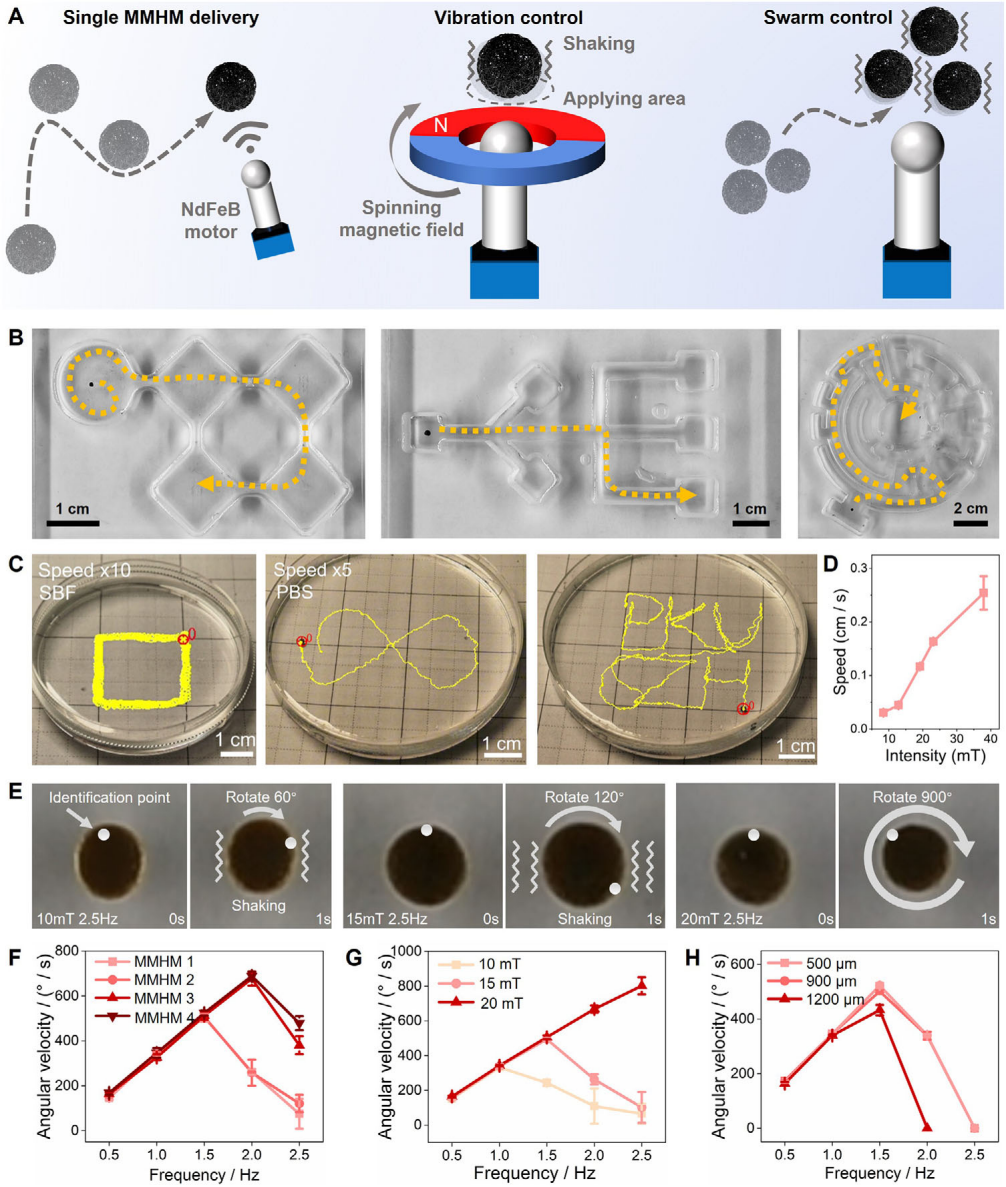
Motion control of MMHMs. A) Schematic diagram shows the single MMHM delivery, vibration control, and swarm control of MMHMs. B) Photographs display the motion trajectories of single MMHM in complex channel models under external magnetic field influence. C) Long‐term locomotion and designated trajectory movement of the MMHMs. D) Movement speed of a MMHM under different magnetic field intensities. E) Vibration control of single MMHM under different magnetic fields. F) Rotational angular velocity of MMHMs with different concentrations of magnetic particles. G) Rotational angular velocity of single MMHM 2 under different magnetic fields. H) Rotational angular velocity of MMHM 2 with different size.

The MMHMs were driven by a self‐made magnetic control system. Under its control, the MMHMs could achieve self‐rotation, vibration, single MMHM directional delivery, and swarm motion (**Figure** **2**A). The directional movement ability of a single MMHM was studied first (Figure 2B). The MMHM has good responsiveness to magnetic fields. Under the driven by a magnetic field with a strength of 15 mT and a frequency of 1.5 Hz, the MMHM could not only move in a straight line, turn, and flip, but also passed through mazes of varying complexity and narrow passes, indicating that the MMHMs have excellent directional delivery potential. The long‐term locomotion and speed modulation of MMHMs under oscillating magnetic fields were systematically investigated. As shown in Figure 2C and Movie S1 (Supporting Information), MMHMs maintained consistent directional movement in phosphate buffer saline (PBS), simulated body fluid (SBF), and saline over extended periods, demonstrating robust structural integrity and resistance to environmental drag forces. Under precise control of the magnetic field, the MMHMs showed excellent controllability, accurately aligning to move along the “infinity symbol” and “PKUSZH” trajectory shapes. Speed analysis (Figure 2D) revealed a linear correlation between magnetic field intensity (8–40 mT) and MMHM velocity, achieving a maximum speed of 0.25 ± 0.03 cm s^−1^ at 37.88 mT. This controllability ensures precise navigation in complex biological environments, critical for targeted delivery to bone defect sites.

Then, the vibration and rotation control of a single MMHM 2 was studied. At a frequency of 2.5 Hz, by controlling the magnetic field intensity, the MMHM 2 can achieve a transition from slight vibration, violent vibration to spin (Figure 2E). The rotation of the MMHM 2 was not always consistent with the rotation frequency of the magnetic control system. This may because when the magnetic field strength decreases or the frequency increases, the response of MMHM to the magnetic field will become weaker, causing the MMHM's speed to be much lower than the speed of the drive motor, and it may even change from rotation to vibration. To better understand the conversion between rotation and vibration, the effects of different magnetic field intensities and frequencies on MMHMs were quantitatively studied (Figure 2F). At a magnetic field strength of 15 mT, MMHM 1 and MMHM 2 began to slow down at frequencies above 1.5 Hz and vibrated at 2.5 Hz, while MMHM 3 and MMHM 4 began to slow down at frequencies above 2 Hz. The MMHM 2 responded well to the rotating magnetic field at a magnetic field strength of 20 mT, with no slowdown or vibration (Figure 2G). At a magnetic field strength of 15 mT and a frequency of 2.5 Hz, the sphere vibrated. At a lower magnetic field strength of 10 mT, the vibration of the MMHM 2 occurred at 2 Hz. The effect of microsphere size on magnetic field responsiveness was also studied (Figure 2H). MMHM 2 slowed down or vibrated at frequencies above 1.5 Hz under a magnetic field strength of 15 mT. The largest‐sized microspheres exhibited the slowest rotation speed and vibrated at lower frequencies.

**Figure 3:**
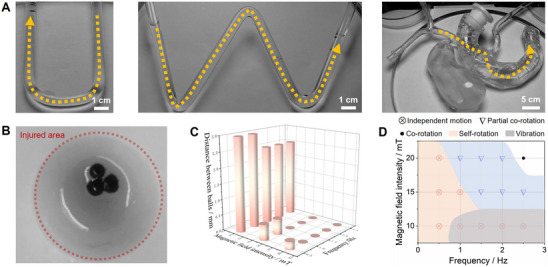
Motion control of MMHM swarm. A) Swarm movement of MMHMs in different models. B) Photograph of MMHMs cluster within an in vitro defect. C) Spacing between MMHMs under different magnetic fields. D) Phase diagram showing movement patterns of MMHM swarm under different magnetic fields.

MMHMs also have good swarm motion performance, which can realize the coordinated motion of multiple MMHMs (**Figure** **3**A). Multiple microspheres can be driven through channels or models of different complexity through the self‐made magnetic control system. The excellent swarm motion ability of MMHMs have a wide range of potential application scenarios. MMHMs of variable sizes and with different concentrations of magnetic particles have various responsiveness under magnetic fields, which gives MMHMs the possibility to repair damage in bone defect areas of various shapes. Repairing a large damage can be achieved by increasing the size or the number of the MMHMs, while repairing defects of different shapes can be achieved by changing the intensity, frequency and direction of the magnetic field. Figure 3B shows the synergistic repair ability of multiple MMHMs. This mechanical stimulation strategy provides ideas for the repair of large bone defects. The frequency and intensity of the driving magnetic field have a decisive influence on the movement mode of the MMHM swarm. Higher frequencies and field strengths would reduce the distance between the MMHMs, causing them to change from individual vibrations or self‐rotations to partial MMHMs rotation around a certain center, and finally to all the MMHMs rotating around the center. In a magnetic field with a field strength of 10 mT and a frequency of 1–3 Hz, the movement mode of the MMHMs was individual vibration (Figure 3C,D). When the frequency dropped below 1 Hz, the MMHMs started self‐spin. Under a field strength of 15 mT and frequency of 0–1 Hz, the MMHMs spun independently while approached to each other. When the frequency increased to more than 1 Hz, some of the MMHMs rotated around a certain center, and the remaining MMHMs kept spinning independently. Under a field strength of 20 mT and a frequency of 0.5 Hz, the MMHMs still spun independently. At a frequency of 1–2 Hz, the MMHMs were close together and some of the MMHMs rotate around a certain center. When the frequency exceeded 2 Hz, they close together to form a whole and rotated around the center.

### 2.3 Theory and Simulation of Magnetic Field and its Effect on MMHMs

The precise control of MMHMs relies on the spatial distribution and gradient of the applied magnetic field. The magnetic field strength **B** and its gradient ∇**B** are critical parameters that determine the force and torque acting on the embedded magnetic particles. For a neodymium magnet modeled as a magnetic dipole, the magnetic field strength at a distance r*r* from the dipole center is expressed as:

(1) $$B\left(r\right)=\frac{\mu_{0}}{4\pi }\cdot \frac{3\left(m\cdot \hat{r}\right)\hat{r}-m}{r^{3}}$$

where **m** is the magnetic moment of the dipole, $\hat{r}$ is the unit vector along *r*, and *µ*_0_​ is the permeability of free space.^[^ ^30^ ^]^ The radial and angular components of **B** exhibit distinct spatial variations, which can be exploited to create controlled gradients for MMHM motion. The rapid decay of **B** with *r* ^3^ necessitates precise positioning of the magnetic source relative to the defect site to maintain sufficient field strength for micromotor actuation.^[^ ^31^ ^]^

The magnetic force **F** *_m_* acting on a single MMHM is proportional to the product of the particle's magnetization **M** and the field gradient ∇**B**:

(2) $$F_{m}=Vp\cdot M\cdot \nabla B$$

where *Vp* is the volume of the magnetic particle. To achieve effective micromotion, the gradient ∣∇**B**∣ must be maximized while maintaining uniformity across the defect site.^[^ ^30^, ^32^ ^]^

**Figure 4:**
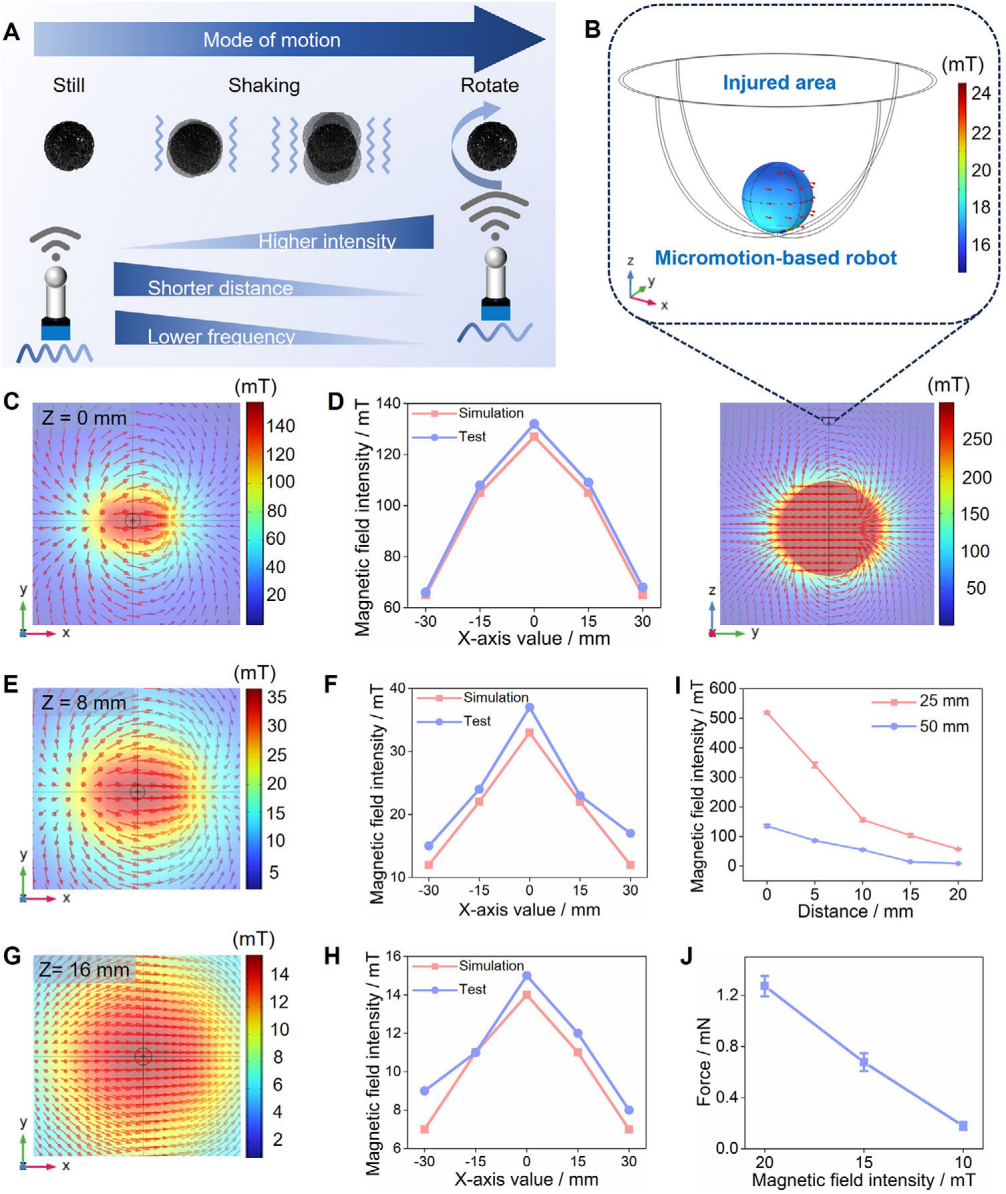
Simulation of in vitro magnetic field intensity and distribution. A) Schematic diagram shows the motion change of the MMHM under varying factors. B) Magnetic field distribution within the injured area and magnetic field MMHM subjected. Simulation of magnetic field distribution and intensity at C) 0mm, E) 8 mm, G) 16 mm. Measured and simulated magnetic field intensity values at D) 0mm, F) 8 mm, H) 16 mm. I) magnetic field intensity generated by NdFeB magnetic spheres of different diameters. J) Generated force of MMHM under different magnetic field intensities.

In addition to translational motion, the rotational dynamics of MMHMs play a pivotal role in their ability to generate localized mechanical stimulation. The torque *τ* exerted on an MMHM is given by:

(3) τ=m×B

where **m** is the magnetic dipole moment of the MMHM. The angular acceleration *α* is governed by the equation of rotational motion:

(4) I·α=τ−ζ·ω

where *I* is the moment of inertia, *ζ* is the rotational drag coefficient, and *ω* is the angular velocity.^[^ ^33^ ^]^ The experimental results (Figure 2F–H) show that the rotational velocity of MMHMs decreases with increasing frequency of the applied magnetic field, indicating a transition from rotation to vibration due to insufficient torque at higher frequencies. This phenomenon is particularly pronounced for larger MMHMs, where increased drag further suppresses rotational motion. At low frequencies (<1.5Hz), the torque is sufficient to overcome drag, resulting in smooth rotation. At higher frequencies (>2Hz), the drag force increases nonlinearly, leading to vibrational motion. The rotational velocity *ω* is inversely proportional to the drag coefficient *ζ*, which scales with the medium's viscosity and the MMHM's size. While the MMHMs are located on the injured site with a curved surface (**Figure** **4**A,B), the localized micromotion induced by MMHMs under the magnetic field generates cyclic mechanical forces that enhance osteoblast activity. The pressure difference Δ*p* created by the micromotion can be estimated as:

(5) Δp=Fm/A

where *A* is the contact area. These forces improve nutrient transport and cellular mechanotransduction, creating a favorable microenvironment for bone regeneration.

Finite element simulations using COMSOL Multiphysics were employed to model the magnetic field distribution and its effects on MMHMs. Figure 4B shows the magnetic field and its distribution generated by a 25 mm diameter NdFeB magnetic ball at a distance of 15 mm from the injured area, and the enlarged image shows the magnitude of the magnetic field to which the MMHM is subjected. Figure 4C,E,G demonstrates the distribution of the magnetic field on the XY 2D plane when the Z value is 0, 8, and 16 mm, where Z = 0 mm represents the outer section of the magnetic sphere, and Z = 16 mm represents the section of the MMHM in the damaged area. The peak value of the magnetic field intensity in contact with the magnetic field source surface is close to 140 mT, which is consistent with the surface field strength characteristics of the permanent magnet, while the magnetic field intensity decays rapidly with the radial distance, which is consistent with the point magnetic dipole model. Figure 4D,F,H are comparison of the simulated magnetic field intensity and the measured magnetic field intensity of the corresponding section, which showed that the simulation has high authenticity and good applicability. The selection of magnetic ball diameter requires a balance between field strength and defect location and size. The magnetic field strength generated by NdFeB magnetic balls with diameters of 25 and 50 mm at different distances was also measured. They can provide magnetic fields with maximum field strengths of about 150 and 500 mT, respectively. When the diameter increases, the magnetic field strength increases significantly, which is consistent with the volume dependence of the magnetic moment (Figure 4I). The mechanical stimulation of the MMHM on the bone injury site is derived from its responsiveness to the magnetic field. Therefore, the positive correlation between the magnetic field strength and the force generated by the MMHM was also quantified (Figure 4J). The force generated by the MMHM increases almost linearly from 0.178 mN at 10 mT to 1.267 mN at 20 mT. The optimization of the force‐field strength relationship provides a theoretical and experimental basis for the precise navigation and energy efficiency improvement of magnetically controlled microrobots in the body.

The theoretical and simulation results provide a mechanistic understanding of how MMHMs enhance bone regeneration. By optimizing the magnetic field parameters (strength, frequency, and gradient) and tailoring the MMHM properties (size, magnetization, and geometry), it is possible to achieve precise control over micromotion and maximize therapeutic efficacy.

### 2.4 In Vitro and In Vivo Biological Properties of the MMHMs

**Figure 5:**
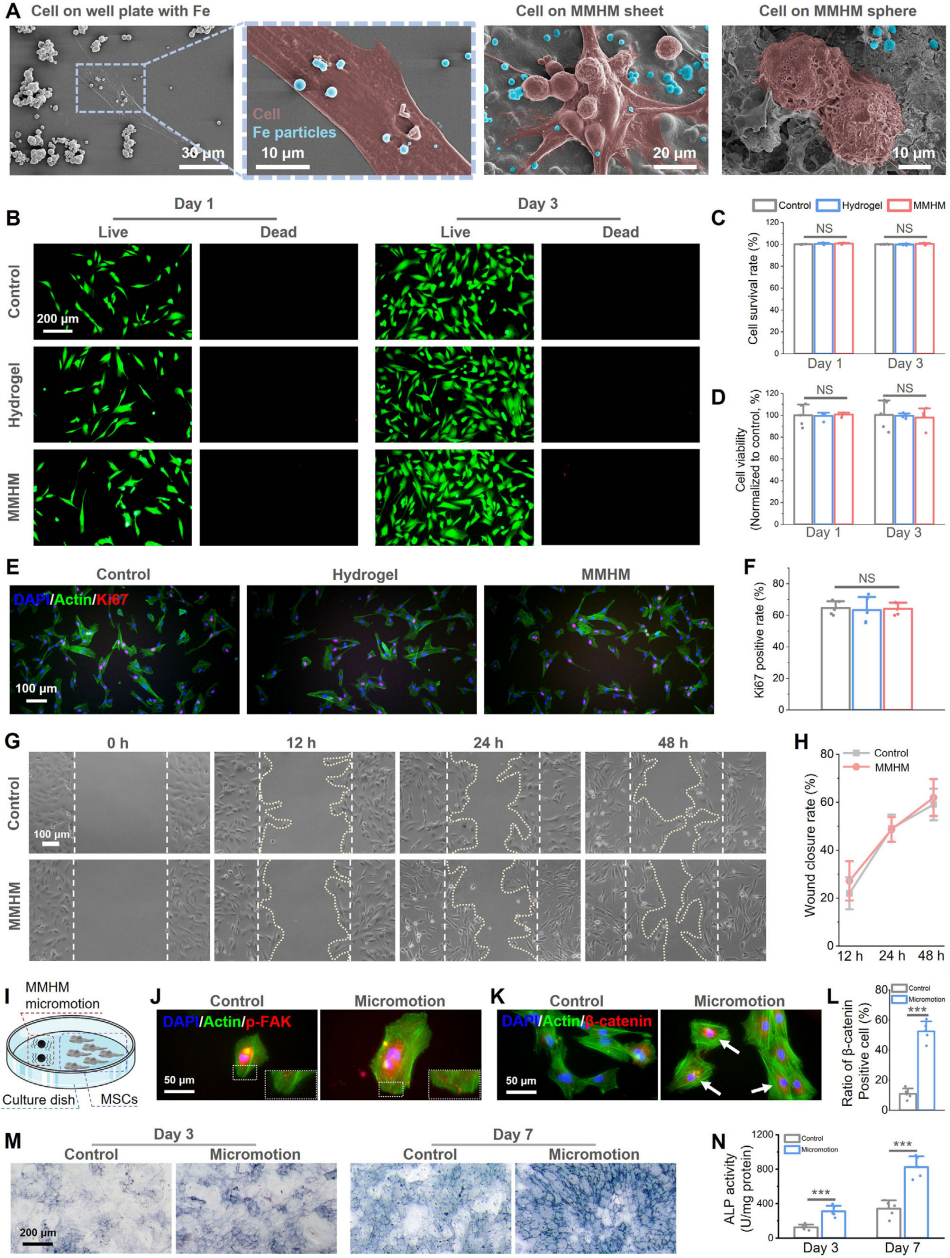
In vitro biological properties of the MMHMs. A) Cell morphologies after culturing on well plate with carbonyl irons (Fe), MMHM sheet, and MMHM sphere for 3 days, where red false color corresponds to cells, and blue false color corresponds to carbonyl irons. B) Live/dead fluorescent staining images and C) quantitative statistics of cell survival rates of mesenchymal stem cells cultured with normal medium (Control), conditional medium of pure hydrogel (Hydrogel) and conditional medium of MMHM (MMHM), where “NS” denotes no significant differences. D) Cell viabilities of mesenchymal stem cells measured by a CCK‐8 assay. E) Immunofluorescence photographs, and F) quantification of the Ki67 expression of the mesenchymal stem cells after cultured with different medium for 1 day, blue (DAPI), green (Actin), and red (Ki67). G) In vitro wound healing of mesenchymal stem cells cultured in different mediums, and H) quantitative analysis of wound closure rate, where the white dashed lines indicate the edge of the original wound area, and light gold dotted curves indicate the edge of cells. I) Schematic illustration of MMHM‐mediated micromotion stimulation on mesenchymal stem cells in a culture dish. J) Representative immunofluorescence images of MSCs stained for nuclei (DAPI, blue), cytoskeleton (Actin, green), and phosphorylated focal adhesion kinase (p‐FAK, red) with/without micromotion stimulation. K) Immunofluorescence images of MSCs stained for nuclei (DAPI, blue), cytoskeleton (Actin, green), and β‐Catenin (red) with/without micromotion stimulation. L) Quantitative analysis of β‐Catenin‐positive MSCs following micromotion stimulation (***p < 0.001 vs static control). M) Alkaline phosphatase (ALP) staining of MSCs after 3‐ and 7‐day osteogenic induction with/without micromotion stimulation. N) Quantitative ALP activity assay of MSCs with/without micromotion stimulation (***p < 0.001). The standard deviation for each bar in this figure was calculated from n = 5 samples.

The cytocompatibility of MMHMs was evaluated using mesenchymal stem cells (MSCs). SEM images (**Figure** **5**A) confirmed normal cell morphology and adhesion on MMHM surfaces, with no cytotoxic effects from carbonyl iron particles. Cytotoxicity is a critical factor to evaluate when assessing the suitability of materials for biomedical implants, as it directly impacts their safety and functionality in clinical applications. In this study, in vitro live and dead cell staining assays demonstrated that neither the pure hydrogel nor the magnetic micromotor hydrogel (MMHM) exhibited any cytotoxic effects on stem cells, regardless of whether the cells were cultured for 1 day or 3 days (Figure 5B,C). This finding indicates that both materials provide a supportive environment for cell survival during short‐term exposure. Furthermore, quantitative cell viability assessments further confirmed the excellent cytocompatibility of both materials (Figure 5D). The observed biocompatibility can likely be attributed to the non‐toxic chemical composition and the hydrophilic nature of the materials, which create a favorable microenvironment for cell attachment, proliferation, and survival. Immunofluorescence staining and quantification of Ki67 (Figure 5E,F) demonstrated 64.02 ± 4.12% MSCs cultured with MMHM‐conditioned medium expressed Ki67, indicating a non‐adverse cellular proliferation. Similarly, in vitro wound healing assays (Figure 5G,H) revealed a compatible migration rate in MMHM‐treated groups compared to control. Notably, the MMHM, despite its functional complexity and the inclusion of magnetic components, maintained high levels of cytocompatibility, underscoring its potential as a safe and effective platform for advanced biomedical applications.

The mechanobiological impact of MMHM‐derived micromotion on MSCs was systematically investigated (Figure 5I–N). Schematic representation (Figure 5I) illustrates the experimental setup where oscillating magnetic fields actuated MMHMs to deliver localized mechanical stimulation to MSC monolayers. This stimulation triggered profound cytoskeletal reorganization, evidenced by enhanced focal adhesion kinase (FAK) phosphorylation at focal adhesion sites (Figure 5J) and pronounced nuclear translocation of β‐Catenin (Figure 5K), hallmarks of Wnt/β‐Catenin mechanotransduction pathway activation. Quantitative analysis confirmed a 4.8‐fold increase in β‐Catenin‐positive cells compared to static controls (*p* < 0.001; Figure 5L), demonstrating mechanosensitive transcriptional regulation. Crucially, micromotion significantly amplified osteogenic commitment, with alkaline phosphatase (ALP) staining revealing dense enzymatic activity (Figure 5M) and ≈2.5‐fold elevations in ALP activity (*p* < 0.001; Figure 5N). These findings establish that MMHM‐generated mechanical forces directly initiate focal adhesion maturation, mechanotransductive signaling, and accelerated osteoblastic differentiation, thereby recapitulating biomechanical cues essential for bone regeneration without exogenous biochemical factors.

**Figure 6:**
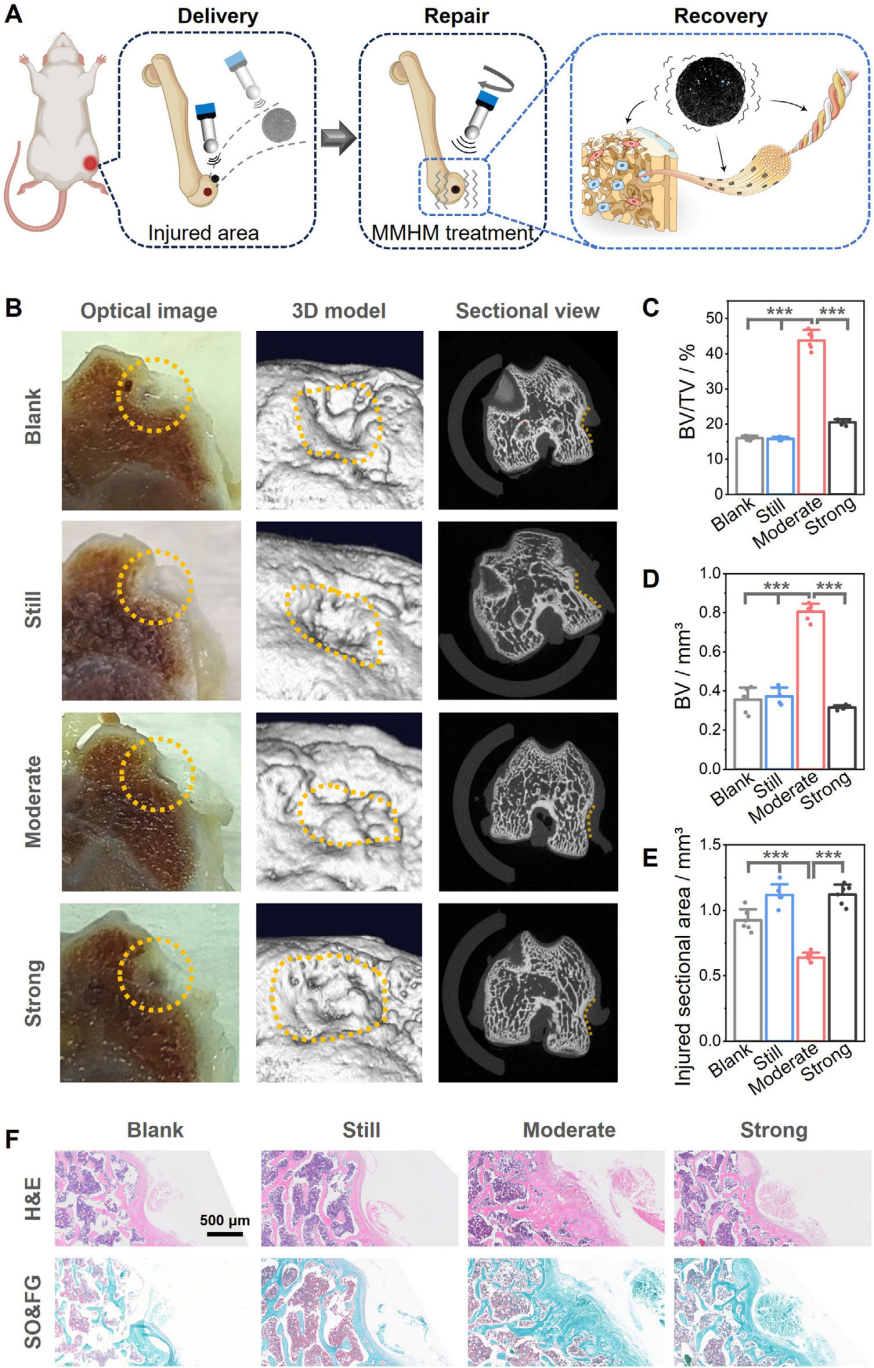
In vivo bone defect repair with the MMHMs. A) Schematic diagram shows the bone repair process with the MMHMs. B) Photographs and micro‐CT reconstructed models of damaged areas, where orange dashed curves denote the defect areas. C) Bone volume fraction, D) bone volume, and E) injured sectional areas within the defects (***p < 0.001). F) Histological analysis of bone formation within the defects stained with hematoxylin and eosin (H&E), safranin O and fast green (SO&FG). The standard deviation for each bar in this figure was calculated from n = 6 samples.

The MMHMs can be driven by the magnetic control system to achieve precise positioning and targeted delivery to the bone defect area, and then promote bone related cell metabolism, collagen production, and calcium deposition through micromotion stimulation, ultimately achieving the effect of promoting bone repair (**Figure** **6**A). To evaluate the effect of MMHMs on bone defect repair under different vibration states, rat femoral defect model was adopted, and micro‐computed tomography (CT) observation and quantitative analysis as well as histological evaluation were used to evaluate the repair effect for one month implantation. The optical images of the distal femoral defect, the Micro‐CT 3D reconstructed models, and the Micro‐CT sectional images are shown in Figure 6B, where the yellow dotted lines are the defect areas. Compared with other groups, the moderate micromotion stimulation group showed excellent osteogenic property, and its bone defect was almost completely repaired (Figure 6B). After the implantation of MMHMs, static or strong mechanical stimulation inhibited osteogenesis, and their amount of new bone formation within the defect were almost the same as that in the blank control group. Micro‐CT quantitative data also supported the results of the images. Figure 6C,D shows the values of bone volume fraction and bone volume of each group. The new bone formation in the moderate stimulation group was significantly higher than that in the other groups. At the same time, its unrepaired area of the bone defect was significantly lower than that in the other groups (Figure 6E). Histological staining was consistent with the above results, showing more new bone formation in the defect of the moderate group (Figure 6F). The density of the new bone tissue was significantly higher than that of the other groups, and the collagen fibers were arranged in an orderly manner. While the current study employed a sub‐critical defect model to evaluate MMHM‐mediated acceleration of natural bone healing, a scenario clinically relevant to stress fractures and small traumatic injuries, it is important to acknowledge that this model does not assess the system's capacity to bridge critical‐sized defects (CSD), which lack inherent regenerative potential. The observed over twofold enhancement in bone volume (Figure 6C) validates MMHMs' ability to amplify endogenous repair through mechanostimulation. However, future studies utilizing CSD models will be critical to fully establish the platform's therapeutic scope for non‐healing defects, a common challenge in severe trauma or osteoporotic fractures.

**Figure 7:**
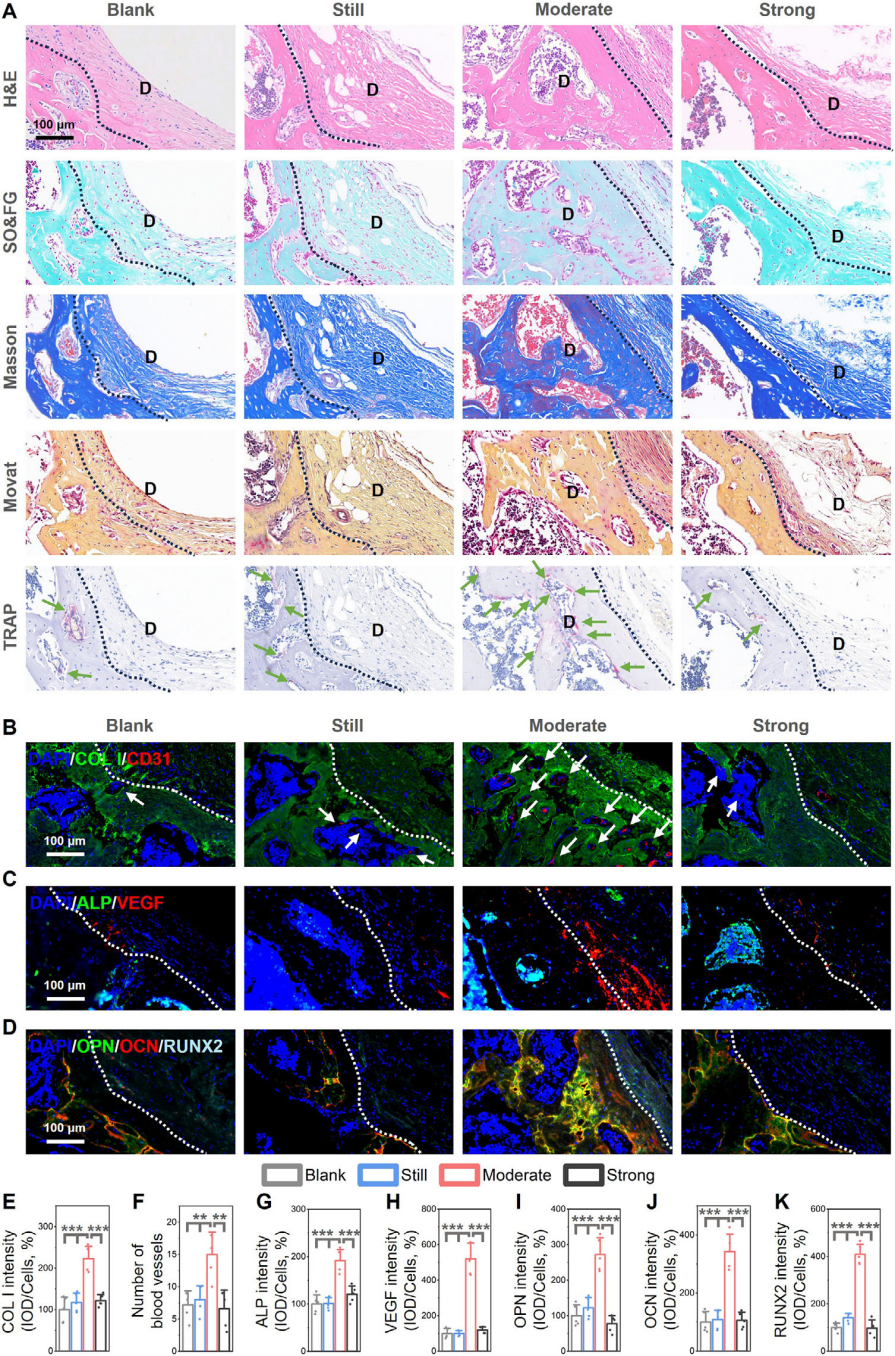
Histological analysis of the MMHM‐repaired bone tissue. A) Histological observation of bone formation within the defects stained with hematoxylin and eosin (H&E), safranin O and fast green (SO&FG), Masson's trichrome, Movat pentachrome, and tartrate‐resistant acid phosphatase (TRAP) staining, where black dotted curves indicate the bone/fibrous connective tissue interfaces, “D” indicates defect area, and green arrows indicate osteoclasts. B) Immunofluorescence photographs, and quantification of E) the type one collagen (COL I) expression and F) number of blood vessels, blue (DAPI), green (COL I), and red (CD31) (**p < 0.01, ***p < 0.001). C) Immunofluorescence photographs, and quantification of G) the alkaline phosphatase (ALP) and H) the vascular endothelial growth factor (VEGF) expression, blue (DAPI), green (ALP), and red (VEGF) (***p < 0.001). D) Immunofluorescence photographs, and quantification of I) the osteopontin (OPN), J) the osteocalcin (OCN) expression, and K) the runt‐related transcription factor 2 (RUNX2) expression, blue (DAPI), green (OPN), red (OCN), and cyan (RUNX2) (***p < 0.001). The white dotted curves indicate the bone/fibrous connective tissue interfaces in the immunofluorescence photographs. The standard deviation for each bar in this figure was calculated from n = 5 samples.

Magnified histological images demonstrated superior bone repair in the moderate‐stimulation group (**Figure** **7**A), characterized by dense collagen alignment and organized mineralization, contrasting with sparse fibrous tissue in still or strong groups. TRAP staining showed increased osteoclast activity in moderate group, further confirming late remodeling stage of bone repair. Immunofluorescence quantification (Figure 7B–K) revealed significant upregulation of osteogenic markers, including COL I (≈2.2‐fold increase, *p <* 0.001), ALP (≈1.9‐fold increase, *p <*0.001), OPN (≈2.7‐fold increase, *p* < 0.001), OCN (≈3.6‐fold increase, *p* < 0.001), and RUNX2 (≈3.9‐fold increase, *p* < 0.001), alongside elevated VEGF expression (≈5.2‐fold, *p <* 0.001), and the number of blood vessels (≈2.1‐fold, *p <* 0.01) indicating enhanced angiogenesis. These data underscore that optimized micromotion stimulates coupled osteogenesis and vascularization, mimicking natural healing cascades.

**Figure 8:**
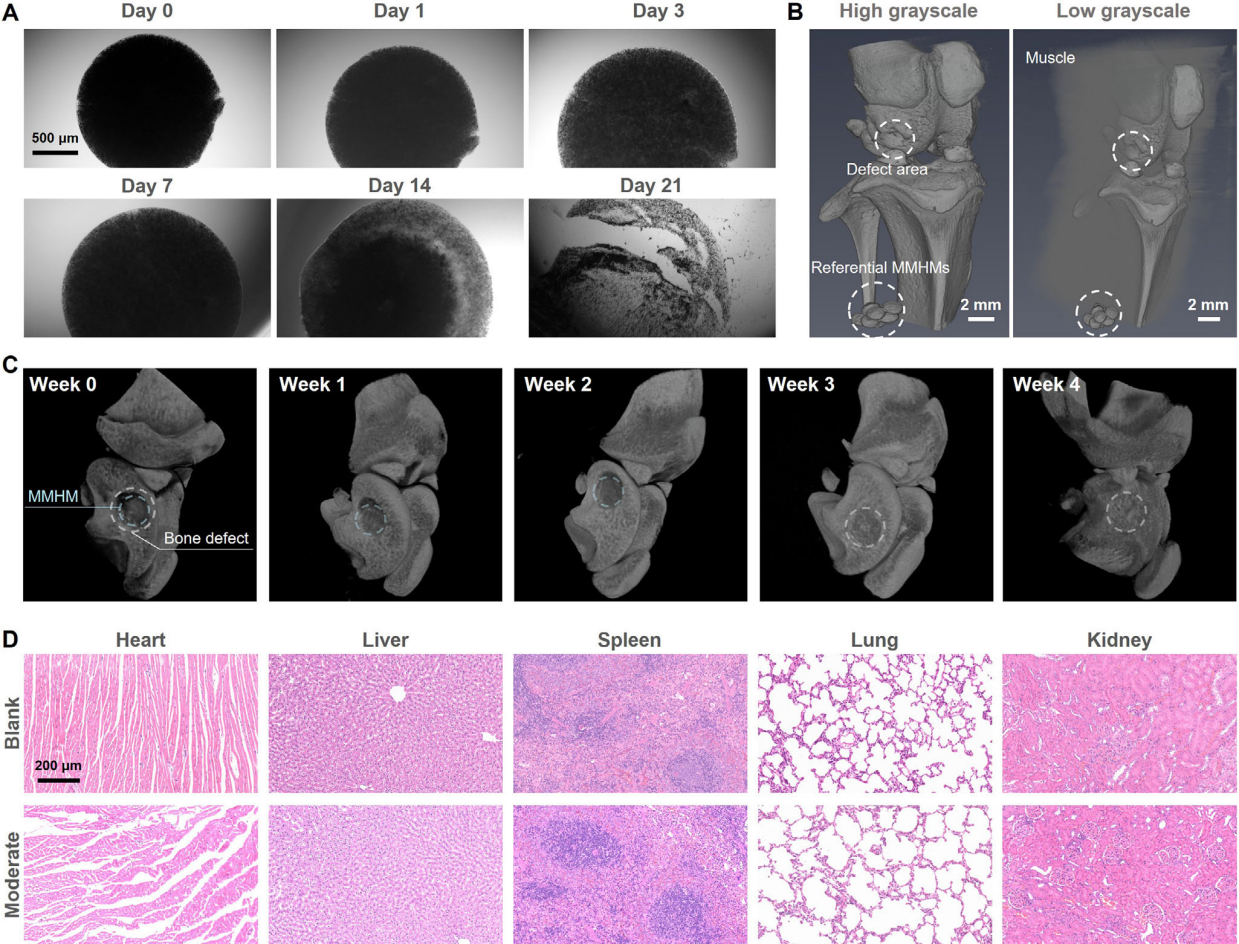
Biodegradation and in vivo biosafety of the MMHMs. A) Biodegradation process of a MMHM after immersed in simulated body fluid (SBF) for 21 days. B) 3D reconstructed Micro‐CT images of referential MMHMs and rat knee joints at different grayscales after a four‐week implantation. C) 3D reconstructed Micro‐CT images of the defect area implanted with MMHMs from week 0 to week 4 post surgery. D) Biosafety of the MMHMs verified by hematoxylin and eosin‐stained heart, liver, spleen, lung, and kidney tissues sections.

The MMHMs exhibited progressive biodegradation in SBF after 21 days of immersion, attributable to hydrogel hydrolysis (**Figure** **8**A). Micro‐CT imaging (Figure 8B) confirmed complete MMHM degradation in vivo, leaving no residual fragments in bone tissues. In vivo biodegradation kinetics were tracked using micro‐CT every week (Figure 8C). MMHMs exhibited progressive volume reduction over 4 weeks, with complete resorption coinciding with defect bridging at Week 4. The degradation rate aligned with bone deposition timelines. The MMHM still exists in the bone defect two weeks after implantation, ensuring sustained mechanical stimulation during critical repair phases. The synchronized degradation kinetics with bone repair timelines ensure sustained mechanical stimulation without long‐term biocompatibility risks, positioning MMHMs as a transient yet effective regenerative platform. Crucially, no residual fragments were detected in neo‐tissue, confirming thorough clearance and absence of long‐term foreign body risks. Histopathological analysis of major organs (Figure 8D) revealed no inflammation or necrosis, as well as diffusion of carbonyl iron particles to major tissues, validating systemic biosafety.

## 3 Conclusion

This study presents a micromotion‐based strategy for bone defect repair using magnetic hydrogel micromotors. Fabricated via microfluidics, MMHMs integrate tunable mechanical properties, superparamagnetism, and biocompatibility, enabling targeted delivery, vibration, and swarm motion under magnetic actuation. Simulations and experiments confirmed a linear correlation between mechanical output and magnetic field strength, providing a foundation for precise control. In vivo studies demonstrated that moderate micromotion significantly enhanced new bone formation (43.89 ± 2.81% bone volume fraction) with organized collagen alignment and more calcium deposition, outperforming static or strong‐stimulation groups. Complete biodegradation and non‐cytotoxicity further support clinical safety. This study establishes an innovative platform for repair of bone defects, expanding the frontiers of magnetic microrobots in regenerative medicine.

## 4 Experimental Section

### Materials

Sodium hyaluronate (Aladdin, 800KDa–1.0 MDa), gelatin (Aladdin, chemically pure), high purity carbonyl iron powder (Guangzhou Metal & Metallurgy (Group) Co., Ltd.), N‐hydroxysuccinimide (NHS, Aladdin, ≥98%), N‐(3‐dimethylaminopropyl)‐N′‐ethylcarbodiimide hydrochloride (EDC, Sigma, ≥97.0%), Span 80 (Aladdin, PharmPure).

### Preparation of Micromotion‐Based Magnetic Hydrogel Micromotors (MMHMs)

A microfluidic platform was employed to prepare the MMHMs. Different concentrations of carbonyl iron powder, 5 mL of 1.33% sodium hyaluronate solution, 5 mL of 20% gelatin solution, 0.033 g of EDC, and 0.033 g of NHS were mixed at 37 °C as the water phase with an injection speed of 300 µL s^−1^, while a mixed solution of paraffin oil and span 80 were used as oil phase with an injection speed of 10 µL s^−1^.

### Characterization of MMHMs

Viscosity of the composite hydrogels was measure by rotational viscometer (NDJ‐1, Lichen Technology) from 30 to 60 °C. Fourier transform infrared scanning (FTIR, Nicolet iN10, Thermo Fisher Scientific), X‐ray Photoelectron Spectrometer (XPS) System (K‐Alpha, Thermo Fisher Scientific) were employed to characterize the structures of the hydrogel and MMHM. The hysteresis characteristics of the samples were measured by 7404 VSM system (Lake Shore Cryotronics). Scanning electron microscopy (SEM, APREO S, Thermo Fisher Scientific) together with energy‐dispersive X‐ray spectroscopy (EDS), and transmission electron microscope (TEM, JEM‐F200, JEOL) were used to observe the structure and element distribution of the materials. Electronic universal testing machine (CMT‐2000, Shandong Meister Industrial Testing System Co., Ltd) was used to measure the mechanical properties of the samples. The hydrogel and the MMHM were immersed in water to measure water absorption with an interval of 0.5 h. Thermostability of the hydrogel and MMHM was measured by a thermogravimetric analyzer (TG 209 F3, Netzsch) with nitrogen atmosphere and a heating rate of 10 °C min^−1^.

### Motion Control of MMHMs

A self‐made test platform and magnetic control system were used to study the effects of different magnetic field environments and parameters on MMHMs. In the single MMHM directional delivery test, the samples were placed in a height‐adjustable maze, and the magnetic control system controlled the movement of the MMHM under different magnetic field strengths. In the single MMHM rotational response test, the samples were placed in a height‐adjustable culture dish with liquid paraffin, and the rotation and vibration of different MMHMs were controlled by adjusting the sample height, the magnetic field strength and frequency of the magnetic control system. In the multi‐MMHM directional delivery, the MMHMs were placed in different pipes or models, and the magnetic control system was adjusted to observe the movement of the MMHMs. In the multi‐MMHM rotational response test, the MMHMs were placed in a height‐adjustable culture dish, and the magnetic field strength and frequency were adjusted to control the movement of the MMHMs.

### Simulation and Verification of Magnetic Field

Comsol Multiphysics 6.2 was used to simulate the magnetic field generated by the NdFeB motor, and a Tesla meter was used to test the magnetic field strength generated by the magnetic motor. The comparison between the simulated magnetic field strength and the actual measured strength was analyzed to confirm the authenticity of the simulation calculation.

### In Vitro and In Vivo Studies

Morphologies of mesenchymal stem cells (MSCs) after culturing on well plate with carbonyl irons (Fe), MMHM sheet, and MMHM sphere for 3 days were observed by using SEM (HT7700, HITACH). After incubation, the specimens were washed by phosphate buffer saline (PBS) for two times and fixed by formalin for 2 h. Next, an up‐grading series of ethanol dehydration were applied to the specimens before air dry. Then, they were coated with platinum in a sputter coater before viewed. For cytocompatibility tests, low glucose DMEM supplemented with 1% P/S and 10% FBS (Control group), medium extract from hydrogel (hydrogel group), and MMHM (MMHM group) that immersed for 24 h were used in this study. The MSCs were seeded onto 96‐well plates at a density of 5.0 × 10^3^ per well. After 1‐ or 3‐days culture, cells were stained with propidium iodide (PI) and calcein‐AM for 30 min, then observed by a fluorescence microscope to assess the cell survival. Cell viability to different materials was evaluated by using a CCK‐8 assay. After 1‐ or 3‐days culture, 10% of the CCK‐8 was added into each well for a 2 h incubation, followed with a measurement using a plate reader. Cell proliferation was further evaluated by Ki67 immunofluorescence staining. After 1 day of culturing, cells were fixed, washed, blocked, incubated with Ki67 primary antibody (Beyotime, 1:100) and secondary antibody (Beyotime, 1:200), counterstained actin (Beyotime, 1:100) and nuclei with DAPI (Beyotime, 1:1000), and observed with the use of fluorescence microscope. An in vitro wound healing assay was employed to exam the effect of different medium on cell migration. After the MSCs were cultured and filled six‐well plates, a 10‐µL pipette tip was used to scratch the confluent cell sheet in a straight line. Then, the medium was changed to normal medium or medium extract from MMHM after PBS washing for two times. A microscope was used to observe the migration of the MSCs, and the wound closure rate was analyzed with ImageJ software. Effects of MMHM‐mediated mechanical stimulation on MSCs were evaluated by immunofluorescence staining and ALP activity detections. The MSCs were seeded and cultured in a culture dish for 24 h followed with vibrational micromotion of MMHMs for 12 h, while static control received no magnetic actuation. Subsequently, cells were fixed for immunofluorescence staining using primary antibodies against phosphorylated focal adhesion kinase (zenbio, p‐FAK; 1:100) and β‐catenin (Servicebio, 1:500), followed by counterstaining with actin (Beyotime, 1:100) and DAPI (Beyotime, 1:1000), and observed with the use of fluorescence microscope. For the ALP staining and activity test, the MSCs were seeded and cultured in a culture dish for 24 h followed with osteogenic induction for 3 and 7 days, and vibrational micromotion of MMHMs for 12 h day^−1^.

The MMHMs were immersed in simulated body fluid (SBF) for 21 days with a refresh of SBF every two days to investigate the biodegradation of the MMHMs. The protocols for animal study adhered to the requirements stated by the Ethics Committee of the Licensing Office of Shenzhen Top Biotechnology Limited Company. Twenty‐four 12‐week‐old female rats were randomly divided into bone defect without material implantation (Control) group, still MMHM after implantation (Still) group, MMHM treated with 1 Hz magnetic field that generate vibrational motion with amplitude around 50 µm (Moderate) group, and MMHM treated with 0.5 Hz magnetic field that undergo rotational motion with angular velocity around 200° s^−1^ (Strong) group. In the distal femur of the rats, 2‐mm diameter defects were drilled and implanted with MMHM for treatment. After 14 days of treatment and 28 days of feeding, the rats were sacrificed at 28 days post‐surgery to collect the femurs followed with a micro‐computed tomography (CT) scanning to evaluate the bone healing within the defects. Fixed samples were decalcified to prepare paraffin sections. The specimens were stained with Hematoxylin and eosin (H&E), Safranin O and Fast Green (SO&FG), Masson's trichrome, Movat pentachrome, and tartrate‐resistant acid phosphatase (TRAP) staining, and histological images were taken by a light microscope. Immunofluorescence staining was performed to further evaluate bone and blood vessel formation with the use of type one collagen (COL I, Servicebio, 1:200), platelet endothelial cell adhesion molecule‐1 (CD31, Servicebio, 1:200), alkaline phosphatase (ALP, Servicebio, 1:200), vascular endothelial growth factor (VEGF, Servicebio, 1:200), osteopontin (OPN, Servicebio, 1:500), osteocalcin (OCN, Servicebio, 1:100), and runt‐related transcription factor 2 (RUNX2, Servicebio, 1:500) anti‐bodies. Heart, liver, spleen, lung, and kidney were harvested, fixed, paraffin‐embedded, sectioned, and stained with H&E to evaluate the biosafety of the MMHMs. To evaluate the degradation of the MMHMs within the defect, fifteen rats were implanted with MMHM and treated with 1 Hz magnetic field that generate vibrational motion. The rats were sacrificed at 0, 7‐, 14‐, 21‐, and 28‐days post‐surgery to collect the femurs followed with a micro‐CT scanning.

### Statistical Analysis

The in vitro experiments were conducted in triplicate. The experimental data were analyzed by a one‐way analysis of variance and expressed as means standard deviations. Tukey multiple comparisons test was used for comparing multiple data sets. Sample sizes were determined via power analysis (target power = 80%, α = 0.05) using g G*Power software to ensure sufficient statistical power. The sample size (n numbers) for each test is shown in the related figure legends. The *p* values are described as follows, * *p* <0.05, ** *p* < 0.01, *** *p* < 0.001, and “ns” denotes not significant.

## Conflict of Interest

The authors declare no conflict of interest.

## Author Contributions

J.S., R.H., and J.H. contributed equally to this work. J.S. and B.W. performed conceptualization; J.S. and B.W. performed methodology; J.S., R.H., J.H., and L.L. performed investigation; B.W. and J.S. performed supervision; J.S. and R.H. wrote the original draft; J.S., B.W., X.F., B.C., Y.H., and S.M. reviewed and edited the manuscript.

## Supporting information



Supplemental Movie 1

## Data Availability

The data that support the findings of this study are available from the corresponding author upon reasonable request.

## References

[1] W. Zhang , H. Ouyang , C. R. Dass , J. Xu , Bone Res. 2016, 4, 15040. 26962464 10.1038/boneres.2015.40 PMC4772471

[2] L. Qin , S. Yang , C. Zhao , J. Yang , F. Li , Z. Xu , X. Hu , Bone Res. 2024, 12, 28. 38744863 10.1038/s41413-024-00332-w PMC11094017

[3] G. L. Koons , M. Diba , A. G. Mikos , Nat. Rev. Mater. 2020, 5, 584.

[4] C. H. Evans , J. Huard , Nat. Rev. Rheumatol. 2015, 11, 234. 25776949 10.1038/nrrheum.2015.28 PMC4510987

[5] G. N. Duda , S. Geissler , S. Checa , S. Tsitsilonis , A. Petersen , K. Schmidt‐Bleek , Nat. Rev. Rheumatol. 2023, 19, 78. 36624263 10.1038/s41584-022-00887-0

[6] B. J. Nelson , S. Pané , Science 2023, 382, 1120. 38060660 10.1126/science.adh3073

[7] T. Li , C. Mao , J. Shen , M. Zhou , Nano Today 2022, 45, 101560.

[8] J. Shen , Y. Wang , M. Yao , S. Liu , Z. Guo , L. Zhang , B. Wang , Matter 2025, 8, 101942.

[9] B. Wang , K. F. Chan , K. Yuan , Q. Wang , X. Xia , L. Yang , H. Ko , Y.‐X. J. Wang , J. J. Y. Sung , P. W. Y. Chiu , Sci. Robot. 2021, 6, abd2813. 10.1126/scirobotics.abd2813 34043547

[10] H. Zhou , C. C. Mayorga‐Martinez , S. Pané , L. Zhang , M. Pumera , Chem. Rev. 2021, 121, 4999. 33787235 10.1021/acs.chemrev.0c01234 PMC8154323

[11] B. Wang , K. Kostarelos , B. J. Nelson , L. Zhang , Adv. Mater. 2021, 33, 2002047. 10.1002/adma.202002047 33617105

[12] F. Barry , M. Murphy , Nat. Rev. Rheumatol. 2013, 9, 584. 23881068 10.1038/nrrheum.2013.109

[13] I. C. Yasa , A. F. Tabak , O. Yasa , H. Ceylan , M. Sitti , Adv. Funct. Mater. 2019, 29, 1808992.

[14] Lu Liu , J. Wu , B. Chen , J. Gao , T. Li , Y. Ye , H. Tian , S. Wang , F. Wang , J. Jiang , J. Ou , F. Tong , F. Peng , Y. Tu , ACS Nano 2022, 16, 6515. 35290021 10.1021/acsnano.2c00833

[15] R. G. Thomas , A. R. Unnithan , M Ju Moon , S. P. Surendran , T. Batgerel , C. H. Park , C. S. Kim , Y. Y. Jeong , Int. J. Biol. Macromol. 2018, 110, 465. 29355634 10.1016/j.ijbiomac.2018.01.003

[16] J. Lee , H.‐W. Song , K. T. Nguyen , S. Kim , M. Nan , J‐Oh Park , G. Go , E. Choi , Micromachines 2023, 14, 434. 36838133 10.3390/mi14020434 PMC9959313

[17] C. Xu , Y. Jiang , H. Wang , Y. Zhang , Y. Ye , H. Qin , J. Gao , Q. Dan , L. Du , L. Liu , Adv. Sci. 2023, 10, 2204881. 10.1002/advs.202204881 PMC9896045 36373692

[18] M. Ding , B. Chen , D. A. Wilson , Y. Tu , F. Peng , Angew. Chem., Int. Ed. 2025, 64, 202423207. 10.1002/anie.202423207 39905915

[19] Q. Li , F. Niu , H. Yang , D. Xu , J. Dai , J. Li , C. Chen , L. Sun , L. Zhang , Adv. Sci. 2024, 11, 2406600. 10.1002/advs.202406600 PMC11578324 39316063

[20] J. Lin , C. Lian , L. Xu , Z. Li , Q. Guan , W. Wei , H. Dai , J. Guan , Adv. Funct. Mater. 2024, 35, 2417146.

[21] X. Wu , L. Zhang , Y. Tong , L. Ren , H. Guo , Y. Miao , X. Xu , Y. Ji , F. Mou , Y. Cheng , J. Guan , ACS Nano 2024, 18, 29558. 39427259 10.1021/acsnano.4c06603

[22] Z. Hu , H. Tan , Y. Ye , W. Xu , J. Gao , L. Liu , L. Zhang , J. Jiang , H. Tian , F. Peng , Y. Tu , Adv. Mater. 2024, 36, 2412227. 10.1002/adma.202412227 39370589

[23] B. Wang , J. Shen , C. Huang , Z. Ye , J. He , X. Wu , Z. Guo , L. Zhang , T. Xu , Nat. Biomed. Eng. 2025, 10.1038/s41551-025-01382-z. 40312457

[24] B. Wang , Q. Wang , K. Chan , Z. Ning , Q. Wang , F. Ji , H. Yang , S. Jiang , Z. Zhang , B. Y. M. Ip , H. Ko , J. P. W. Chung , M. Qiu , J. Han , P. W. Y. Chiu , J. J. Y. Sung , S. Du , T. W. H. Leung , S. C. H. Yu , L. Zhang , Sci. Adv. 2024, 10, adk8970. 10.1126/sciadv.adk8970 PMC10830105 38295172

[25] G. Go , S.‐G. Jeong , A. Yoo , J. Han , B. Kang , S. Kim , K. T. Nguyen , Z. Jin , C.‐S. Kim , Y. R. Seo , J. Y. Kang , J. Y. Na , E. K. Song , Y. Jeong , J. K. Seon , J.‐O. Park , E. Choi , Sci. Robot. 2020, 5, aay6626. 10.1126/scirobotics.aay6626 33022593

[26] G. Go , J. Han , J. Zhen , S. Zheng , A. Yoo , Mi‐J Jeon , J‐Oh Park , S. Park , Adv. Healthcare Mater. 2017, 6, 201601378. 10.1002/adhm.201601378 28481009

[27] G. Go , A. Yoo , H.‐W. Song , H‐Ki Min , S. Zheng , K. T. Nguyen , S. Kim , B. Kang , A. Hong , C.‐S. Kim , J‐Oh Park , E. Choi , ACS Nano 2021, 15, 1059. 33290042 10.1021/acsnano.0c07954

[28] M. Cao , R. Sheng , Y. Sun , Y. Cao , H. Wang , M. Zhang , Y. Rui , Nano‐Micro Lett. 2024, 16, 251. 10.1007/s40820-024-01464-8 PMC11263536 39037551

[29] Li G. , Li Z. , Min Y. , Chen S. , Han R. , Zhao Z. , Small 2023, 19, 2302927. 10.1002/smll.202302927 37264732

[30] E. Marsden , T. J. R. Hughes , Mathematical Foundations of Elasticity, Dover Publications, New York 1994.

[31] P. Tierno , R. Golestanian , I. Pagonabarraga , F. Sagués , J. Phys. Chem. B 2008, 112, 16525. 19367983 10.1021/jp808354n

[32] K. Peyer , L. Zhang , B. Nelson , Nanoscale 2013, 5, 1259. 23165991 10.1039/c2nr32554c

[33] D. G. Grier , Nature 2003, 424, 810. 12917694 10.1038/nature01935

